# Mr. Ring's Cases of Secondary Small-Pox

**Published:** 1807-10

**Authors:** John Ring

**Affiliations:** New-Street, Hanover-Square


### *To the Editors of the Medical and Physical Journal.*

GENTLEMEN,

MANY instances of the Small-pox occurring a second time in the same person, have of late been published, and authenticated as well as any medical facts can be. In addition to those which I have already given in your Journal, and in my other publications, I now beg leave to state the following.

A son of the late Henry Elliston, Esq. of Whitehaven, was inoculated with the small-pox by a surgeon at Egremont, and had a good crop; nevertheless, I am assured by a lady of great respectability, that he has since been attacked again with the disorder, and had it severely.

I have lately seen a letter from Mr. Shrapnell, surgeon of the South Glocester Militia, to Dr. Jenner, in which he relates several instances of a similar kind; particularly one which occurred in the wife of John Alder, a musician in the regiment, who had the small-pox twice, and was blind with it both times.

Mr. Shrapnell observes, that the occurrence of the small-pox a second time is no new idea: John of Gaddeston, physician to Edward the First, says, in his Rosa Anglica, *Homo variolatur bis.*

The following case was communicated to me by Mrs. Stone, housekeeper to Viscount Templetown. Susannah Piper, of Sudycamps, Cambridgeshire, had the small-pox by inoculation; and was blind three days. She some years afterwards nursed Mrs. Stone, who is her sister, and her brother, when under inoculation; in consequence of which she again had a considerable eruption of the small-pox in all parts of the body; and was in all respects more ill than either of the patients whom she attended.

Mrs. Quartermain, 13, Salisbury Place, had the small-pox 26 years ago. She was blind, and so ill that she was given given over by two medical men. Four years ago, when nursing her daughter in the disease, she had an eruption of about fifty pustules, with great constitutional indisposition for several days. She had violent sickness, pain in her head and back; and every other symptom that commonly attends the disorder.

Ann Tolhurst, late servant to Mr. Courtenay, of Lincoln's Inn Fields, was inoculated by Mr. Watts, of Cranbrook. Her arm rose; she was very ill, and had eruptions. Thirteen years after, she nursed her brother's children in the small-pox; in consequence of which she caught the disorder again. She had a head-ach, sickness and fever; followed by so considerable an eruption, that people were terrified, and ran from her, when they met her in the street.

Mrs. Palmer, No. 25, Green Walk, Surry Road, many years after she had had the small-pox, nursed a child of the Honourable Robert Anstruther, when labouring under that disorder; in consequence of which, she had about forty pustules on her breast and shoulder. She was sick, feverish, and had a pain in her head. She was at Mr. Batsford's in Brunswick Place, when I went with the Rev. Mr. Hill to see the case of failure in his family; and she declared, that she had the disorder a second time much more severely than Mr. Batsford's child had it after vaccination.

Mrs. Martin, No. 6, Brunswick Place, was inoculated for the small-pox by Mr. Read, of Portshead, near Bristol; and had a heavy crop. Six years after, she was in the house where the disorder was; in consequence of which she again caught the disease; and had eight or nine hundred pustules, attended with all the symptoms that usually attend the natural small-pox. The pustules began to turn on the seventh day.

The Editors of the Bibliotheque Britannique, in the 28th vol. of that work, p. 64, assent to the truth of this opinion, in the following words: "It is well known, that it is possible to have the small-pox twice; as a great number of respectable authors attest."

---

Since most of the preceding remarks were written, your Journal for September has appeared; in which Mr. Davies, of Piccadilly, expresses his opinion, that *de mortuis nil nisi bonum* is a good motto; I beg leave, however, to express my opinion, that *de mortuis nil nisi verum* is a better.

I am ready to admit, that the work of a deceased author should be suffered to rest in peace, unless it can be shewn, that justice to the living, and to posterity, demands an investigation of its contents. I also admit, that in the course of such investigation every man ought to be cool and temperate; if, therefore, in continuing my inquiry into the truth of Dr. Rowley's publication, I should be betrayed into an improper warmth by the mis-representations which it contains, I trust, the editors, in their wisdom, will expunge the passage.

The Royal College of Physicians have done much towards dispelling the doubts of the public; but I am assured from various quarters, that the unfavourable cases which have been published still have great weight with the multitude, whom it is our duty to undeceive. The remaining cases, in the pamphlet alluded to, which I shall deem it neccssary to refute, are not numerous; and I hope no reader of your Journal will have reason to complain of the space which this refutation will occupy.

Two children of Mr. Colbeck, No. 3, Portland Street, Soho, are stated by Dr. Rowley to have had the small-pox after the cow-pock; but Mr. and Mrs. Colbeck assured me, that the eruption first appeared in the form of blisters, and was supposed to be the chicken-pox.

In several of the other cases of pretended failure, which I have inquired into, the second disorder was the same as in the preceding instance; in some the patients were previously infected, and in others, the subsequent small-pox was merely local.

The child of John Meredith, of Kensington, who died of the small-pox, is stated by Dr. Rowley to have been vaccinated in Great Castle Street, Oxford Market; he was, however inoculated in Castle Street, Leicester Square; and I have already proved in your Journal, that he did not go through vaccination in a regular way, the pustules being destroyed at an early period of the disease.

The child of Mr. Turtle, in Oxford Street, near Argyle Street, is said by Dr. Rowley to have had the cow-pox mange. He says, the case was treated unsuccessfully as the itch. Cases of the itch, it is well known, are unsuccessfully treated, from various causes. In the present, the child was under the care of Dr. Merriman; who declares, that if he ever saw the itch in his life, this was the itch. No one will suppose it was *unskilfully* treated by him.

That the disorder was not occasioned by the cow-pox is evident to every candid inquirer; for it appears from the mother's mother's account, that it did not commence till seven months after vaccination; when the child was put out to be weaned, and slept with a servant out of place, whose subsequent conduct is sufficient to warrant an opinion, that she might have had the itch.

The child of Mr. Mazoyer, of Grafton Street, is said by Dr. Rowley to have had the small-pox, of which he died, after vaccination. To give this case the greater authenticity, he says, "sent by Dr. Coombe's, Bloomsbury, to see the case, in company with Dr. Kennedy. Seen by many of the faculty." This instance furnishes a specimen of the manner in which many of the faculty inquired into such cases; for when I asked Mrs. Mazoyer, whether any mark remained after vaccination, she replied, "there was a mark at first; but it has not been visible for the last six months."—This is a sufficient proof, that the child was not properly vaccinated.

Dr. Rowley says, "the tragic scene would have melted a soul of adamant; though a cruel vaccinator abused the parents in their deep affliction."—This is one of the foulest calumnies on record; and no friend of vaccination, or of justice and truth, can wish it to remain unrefuted. Mrs. Mazoyer assures me, that every person who came to her house on this occasion, behaved with the utmost humanity and politeness; except one, who is a quack, and of course an anti-vaccinist. This man spoke in a very rude manner to one of the friends of vaccination; who, as Mrs. Mazoyer observes, excused it on account of the quarter whence it came."

In one respect, Dr. Rowley is perfectly right in his assertion. He says, "the indefatigable Dr. Moseley pursues the subject, and has favoured the author with a succession of disastrous events from vaccination, enough to fill the soul with horror; and all *credulous* parents with the most pungent grief."

It would be an endless task to point out all the misrepresentations in this work; but it is proper to observe, that every kind of exaggeration occurs in almost every page. One child is said to have been in danger of losing its arm, who had only a common abscess; and others, from whom drawings were made, are said to have fallen victims to the small-pox, although they have never had that disorder; and I have reason to believe, that they are at this moment in perfect health.

Such are the cases, for the truth of which, or at least of a considerable proportion of them, Mr. Birch contends, in in his "Serious Reasons."—This is a serious reason, in addition to others, why the fallacy of those cases should be pointed out.

There is another fallacy, which ought also to be pointed out, uamely, the mean artifice of republishing old works under a new title, which has several times been practised by the anti-vaccinists; and which, as is observed by certain reviewers, in their remarks on Mr. Birch's pamphlet, may be considered as *a take-in.* On that pamphlet, as well as Dr. Moseley's "Oliver for a Rowland," I intend shortly to publish some remarks in another form.

Before I conclude this letter, it may be proper to make some remarks on one or two other passages of Mr. Davies's communication. He says, when new theories in medicine are discovered, they will be received by experienced practitioners with caution, and examined with candor. Here he probably alludes to the candour of his neighbour, the experienced practitioner at the Albany; who condemned vaccination before he saw it, or knew what it was.

Mr. Davies insinuates, that a subject of *controversy* cannot be of a *practical* nature. This is no wonder, when he considers the discovery of vaccination only as the discovery of a new theory in medicine. But perhaps he may say, and say without any great violation of truth, *Davus sum, non Œdipus.*

He says the practice is sanctioned by general approbation, throughout the greatest part of the habitable globe; and therefore thinks it unnecessary to "*Ring*" another peal in the ears of the medical world on that long debated point. But I beg leave to observe, that it is not at present sanctioned with general approbation in this country, owing to the obstacles thrown in its way by certain obscure practitioners; some of whom are its *pretended* friends.

Mr. Davies justly remarks, that there is still room for improvement in the healing art. I cannot, however, consider that man a friend to improvement, who wishes to prevent a full investigation of what comes from the press.—Vaccination is undoubtedly a *practical* art; and were it a *profitable* one also, it would have met with less opposition.

I am, &c.

JOHN RING.

